# Prolactin blocks the expression of receptor activator of nuclear factor κB ligand and reduces osteoclastogenesis and bone loss in murine inflammatory arthritis

**DOI:** 10.1186/s13075-017-1290-4

**Published:** 2017-05-15

**Authors:** Maria G. Ledesma-Colunga, Norma Adán, Georgina Ortiz, Mariana Solís-Gutiérrez, Fernando López-Barrera, Gonzalo Martínez de la Escalera, Carmen Clapp

**Affiliations:** Instituto de Neurobiología, Universidad Nacional Autónoma de México (UNAM), Campus UNAM-Juriquilla, 76230 Querétaro, México

**Keywords:** Hormones, Bone, Inflammation, Experimental arthritis, Osteoclasts, Synovial fibroblasts, Prolactin receptor, RANKL, RANK

## Abstract

**Background:**

Prolactin (PRL) reduces joint inflammation, pannus formation, and bone destruction in rats with polyarticular adjuvant-induced arthritis (AIA). Here, we investigate the mechanism of PRL protection against bone loss in AIA and in monoarticular AIA (MAIA).

**Methods:**

Joint inflammation, trabecular bone loss, and osteoclastogenesis were evaluated in rats with AIA treated with PRL (via osmotic minipumps) and in mice with MAIA that were null (*Prlr*-/-) or not (*Prlr*+/+) for the PRL receptor. To help define target cells, synovial fibroblasts from *Prlr*+/+ mice were treated or not with proinflammatory cytokines ((Cyt), including TNFα, IL-1β, and interferon (IFN)γ) with or without PRL, and these synovial cells were co-cultured or not with bone marrow osteoclast progenitors from *Prlr*+/+ or *Prlr*-/- mice.

**Results:**

In AIA, PRL treatment reduced joint swelling, increased trabecular bone area, lowered osteoclast density, and reduced mRNA levels of osteoclast-associated genes (tartrate-resistant acid phosphatase (*Trap*))*,* cathepsin K (*Ctsk*)*,* matrix metalloproteinase 9 (*Mmp9*)*,* and receptor activator of nuclear factor κB or RANK (*Tnfrsf11a*)), of genes encoding cytokines with osteoclastogenic activity (*Tnfa, Il1b, Il6,* and receptor activator of nuclear factor κB ligand or RANKL (*Tnfrsf11*)), and of genes encoding for transcription factors and cytokines related to T helper (Th)17 cells (*Rora, Rorc*, *Il17a*, *Il21*, *Il22*) and to regulatory T cells (*Foxp3*, *Ebi3*, *Il12a*, *Tgfb1*, *Il10*). *Prlr-/-* mice with MAIA showed enhanced joint swelling, reduced trabecular bone area, increased osteoclast density, and elevated expression of *Tnfa*, *Il1b*, *Il6*, *Trap*, *Tnfrsf11a*, *Tnfrsf11*, *Il17a*, *Il21*, *Il22*, *1 l23*, *Foxp3*, and *Il10.* The expression of the long PRL receptor form increased in arthritic joints, and in synovial membranes and cultured synovial fibroblasts treated with Cyt. PRL induced the phosphorylation/activation of signal transducer and activator of transcription-3 (STAT3) and inhibited the Cyt-induced expression of *Il1b, Il6,* and *Tnfrsf11* in synovial fibroblast cultures. The STAT3 inhibitor S31-201 blocked inhibition of *Tnfrsf11* by PRL. Finally, PRL acted on both synovial fibroblasts and osteoclast precursor cells to downregulate Cyt-induced osteoclast differentiation.

**Conclusion:**

PRL protects against osteoclastogenesis and bone loss in inflammatory arthritis by inhibiting cytokine-induced expression of RANKL in joints and synovial fibroblasts via its canonical STAT3 signaling pathway.

**Electronic supplementary material:**

The online version of this article (doi:10.1186/s13075-017-1290-4) contains supplementary material, which is available to authorized users.

## Background

Rheumatoid arthritis (RA) is an immune-mediated type of inflammatory arthritis that causes massive bone destruction by increasing osteoclast development (osteoclastogenesis) and activity. Receptor activator of nuclear factor κB ligand (RANKL), its cellular receptor, receptor activator of nuclear factor κB (RANK), and the decoy soluble RANKL receptor osteoprotegerin (OPG) are key regulators of osteoclastogenesis in RA [1–3]. RANKL is produced by osteoblastic lineage cells, activated T cells, synovial fibroblasts, and other stromal cells, and it binds to RANK on osteoclast precursors to stimulate their differentiation, activity, and survival [4–8]. The effects of RANKL are neutralized by OPG which, by binding to RANKL, prevents it from activating RANK. RANKL and OPG are pivotal downstream signals upon which many cytokines, growth factors, and steroid and peptide hormones converge to regulate bone homeostasis [7, 8]. Shifting the RANKL-to-OPG ratio in favor of bone protection is a promising therapeutic strategy against joint destruction in RA. Prolactin (PRL), the hormone essential for mammary gland development and milk production, may have this protective effect.

The female preponderance and the influence of reproductive states in RA, together with PRL immune-enhancing actions, have long linked this disease to a detrimental effect of PRL [9–11]. However, accumulating evidence has challenged this view by showing that PRL is also immunosuppressive [12–14], and that hyperprolactinemia occurring during pregnancy and lactation [15, 16] or induced pharmacologically by the dopamine D2 receptor antagonist haloperidol [17], is associated with a reduction in the severity and risk of RA. Moreover, increasing prolactinemia by PRL infusion or treatment with haloperidol ameliorates inflammation and joint destruction in AIA as revealed by reduced joint swelling, lower local expression of proinflammatory cytokines TNFα, IL-1β, IFNγ, and IL-6, and inhibition of chondrocyte apoptosis, pannus formation, and bone erosion [18].

PRL protection against bone loss in inflammatory arthritis is not unexpected. PRL-receptor-null mice are osteopenic [19] and PRL treatment increases bone formation during growth by decreasing the RANKL/OPG ratio in osteoblasts [20]. Moreover, PRL stimulates the proliferation of pancreatic β cells by promoting OPG-mediated inhibition of RANKL [21], and RANKL is under the control of PRL to promote mammary gland lobulo-alveolar development during pregnancy [22].

Here, we investigated whether PRL, either by its exogenous administration or by the genetic deletion of the its receptor, reduces trabecular bone loss and osteoclastogenesis by modifying proinflammatory cytokine-induced upregulation of the RANKL/RANK/OPG system in rodent polyarticular AIA and monoarticular AIA (MAIA), and whether these actions involve a direct effect of PRL on synovial fibroblasts and osteoclast progenitor cells.

## Methods

### Animals

Male Sprague-Dawley rats (200–250 g) and female C57BL6 mice, wild type (*Prlr*+/+*)* or null for the PRL receptor (*Prlr*-/-*)* (8 weeks old, 20–25 g), were housed under standard laboratory conditions (22 °C; 12-hour/12-hour light/dark cycle; free access to food and water).

### Induction of AIA

AIA was induced in rats as described [18], by a single intradermal injection at the base of the tail of 0.2 ml complete Freund’s adjuvant (CFA, Difco Laboratories, Detroit, MI, USA; 10 mg heat-killed *Mycobacterium tuberculosis* per 1 ml of Freund’s adjuvant). Three days before CFA injection some rats were rendered hyperprolactinemic by the subcutaneous implantation of a 28-day osmotic minipump (Alza, Palo Alto, CA, USA) containing 1.6 mg of ovine PRL (Sigma Aldrich, St. Louis, MO, USA). Ankle swelling, monitored as described [18], indicated that the onset of arthritis appeared on day 12 after CFA injection and was maximal by day 21, when the animals were euthanized, serum samples collected for PRL and cytokine determinations, and ankle joints processed for histological examination, quantitative (q)PCR, and western blot.

### Induction of MAIA

Monoarticular AIA was induced and assessed in mice as described [23]. Briefly, mice were injected into the articular space of the right knee joint with CFA (5 μg in 10 μl) once every 7 days for 18 days. The diameter across the knee joint (left and right) was measured twice a week with a micro-caliper. Mice were euthanized 18 days after the initial CFA injection, and knee joints were removed and processed as above.

### Intra-articular injection of TNFα, IL-1β, and IFNγ (Cyt)

Mice were injected in the articular space of the knee joints with Cyt in a final volume of 20 μl (62.5 ng TNFα, 25 ng IL-1β, and 25 ng IFNγ; R&D Systems, Minneapolis, MN,USA) or with endotoxin-free water as vehicle. Forty-eight hours after Cyt or vehicle injection, mice were euthanized and synovial membranes were extracted and processed for quantitative real-time (qRT)-PCR evaluation.

### Serum measurements

Infused ovine PRL was measured in serum by the Nb2 cell bioassay, a standard procedure based on the proliferative response of the Nb2 lymphoma cells to PRL, carried out as described [18]. The serum levels of C-reactive protein and TNFα were quantified using ELISA kits from BD Biosciences (San Jose, CA, USA) and R&D systems (Minneapolis, MN, USA), respectively.

### Histological examination and image analysis

Ankle and knee joints were fixed, decalcified, and dehydrated for paraffin embedding. Four 7-μm-thick sections spaced 380 μm or 126 μm apart, per each rat tibia/tarsal joint or mouse femur/tibia joint, respectively, were stained with Harris’s hematoxylin-eosin solution for measurement of trabecular bone area and with tartrate-resistant acid phosphatase (TRAP) for evaluation of osteoclast number. For the latter, deparaffinized sections were washed, and incubated for 3 hours at 37 °C in TRAP activity staining mix (Fast Red Violet LB Salt (80 mg; Sigma Aldrich), Naphtol AS-MX (40 mg; Sigma Aldrich), formamide (4 ml; Invitrogen, Carlsbad, CA, USA), 0.04 M sodium acetate (0.656 g), 0.2 M disodium tartrate dihydrate (9.2 g), and distilled water (200 ml)). The pH was adjusted to 5.0 and pre-incubated to 37 °C before use. After incubation, sections were rinsed with distilled water, counterstained with Mayer’s hematoxilin (Sigma Aldrich), and coverslipped with mounting medium (Entellan, Merck Millipore Corporation, Billerica, MA, USA). Hematoxylin-eosin-stained and TRAP-stained tissue sections were visualized by light microscopy (Olympus BX60F5, Olympus Tokyo, Japan). Trabecular bone surface and number of TRAP-stained purple spots (osteoclasts) were quantified (Image-Pro Plus analysis software; Media Cybernetics, Silver Spring, MD, USA) and divided by total bone area to obtain trabecular bone area and osteoclast density. Two independent observers, blind to the experiments, performed the measurements.

### qRT-PCR

Frozen whole ankle and knee joints were pulverized in liquid nitrogen using a mortar and pestle. Total RNA was isolated using TRIzol reagent (Invitrogen, Carlsbad, CA, USA) and reverse transcribed using the High-Capacity cDNA Reverse Transcription Kit (Applied Biosystems, Foster City, CA, USA). PCR products were detected and quantified using Maxima SYBR Green qPCR Master Mix (Thermo Fisher Scientific, Waltham, MA, USA) in a final reaction of 10 μl containing template and 0.5 μM of each of the primer pairs for different genes (see Additional file 1: Table S1). Amplification performed in the CFX96 real-time PCR detection system (Bio-Rad, Richmond, CA, USA) included a 10-minute denaturation step at 95 ° C, followed by 35 cycles of amplification (10 sec at 95 °C, 30 sec at the primer pair-specific annealing temperature, and 30 sec at 72 °C). The PCR data were analyzed by the 2-^ΔΔCT^ method, and cycle thresholds (CT) normalized to the housekeeping gene hypoxanthine-guanine phosphoribosyltransferase (*Hprt)* were used to calculate the mRNA levels of interest.

### Isolation and culture of synovial fibroblasts

Synovial fibroblasts were isolated from the hind limbs of wild-type mice separated at the femur/fibula/tibia junctions, as previously described [24]. Briefly, limbs were washed in Hanks’ balanced salt solution (HBSS), and dissected while immersed in high glucose DMEM (Invitrogen, Carlsbad, CA, USA) supplemented with 20% heat-inactivated fetal bovine serum (FBS; Gibco, Grand Island, NY, USA) and antibiotics (50 U/ml of penicillin and 50 μg/ml of streptomycin; Invitrogen) to remove all soft tissues from bones. The joint space was opened with a scalpel to expose the synovial tissues and incubated in culture medium containing 1 mg/ml collagenase type IV (Difco Laboratories) and 0.1 mg/ml of deoxyribonuclease I (Sigma Aldrich) in a shaking bath for 3 hours at 37 °C. Tissues were then vortexed vigorously to release cells. The supernatant was passed through an 80-μm filter and centrifuged; cells were re-suspended in fresh culture media and grown to confluency. Cells were used for experiments after 2–4 passages, when the cultures showed >98% synovial fibroblast phenotype (CD90.2+, VCAM1+, and ICAM-1+) as described [24].

Synovial fibroblasts were seeded at 10^6^ cells/well in 6-well plates and incubated in 2 ml of culture medium for 16 hours with or without PRL (100 nM, recombinant human PRL provided by Michael E. Hodsdon, Yale University, New Haven, CT, USA). Cyt (0.25 ng/ml TNFα, 0.05 ng/ml IL-1β, and 0.05 ng/ml IFNγ) were then added or not to the culture for an additional period of 24 hours. Other cell cultures were pre-incubated for 2 hours with or without the STAT inhibitor, S31-201 at 50 nM (Santa Cruz Biotechnology, Santa Cruz, CA, USA), and then incubated or not with 100 nM PRL for 16 hours, followed by the treatment with or without Cyt for 24 hours. To evaluate PRL effects on phosphorylation/activation of STAT3, synovial fibroblasts were seeded in complete medium for 24 hours, cultured in serum-free medium for another 24 hours, and then pre-treated for 6 hours with the Cyt followed by a 30-minute incubation in the presence or absence of Cyt with or without 100 nM PRL.

### Co-culture of synovial fibroblasts and osteoclast progenitors

Synovial fibroblasts (passage 4) were seeded at 5 × 10^5^ cells in 48-well plates and after 12 hours the cells were treated or not with PRL for 16 hours. Cyt were then added or not to the culture, and after 24 hours, bone marrow cells (2 × 10^6^) containing osteoclast progenitors, harvested from the tibias and femurs of 8-week-old C57BL/6 mice, wild type (*Prlr+/+*) or null for the PRL receptor (*Prlr-/-*), were delivered in culture medium with 1, 25 dihydroxy-vitamin D3 (10^-8^ M, Sigma-Aldrich). Co-cultures were incubated for 10 days changing for new medium (containing 1, 25 dihydroxy-vitamin D3, +/- Cyt, +/- PRL) every 2 days. Co-cultures were fixed with 3.7% formaldehyde for 10 minutes at room temperature, washed twice with PBS, air-dried, and incubated for 20 minutes at 37 ^o^C with TRAP activity staining mix. Osteoclastogenesis was assessed by counting the number of enlarged TRAP-positive (TRAP+) cells.

### Isolation of chondrocytes, osteoblasts, and osteoclast-like cells

To identify other joint cells expressing the PRL receptor, chondrocytes, osteoblasts, and osteoclast-like cells were obtained from *Prlr+*/*+* and *Prlr*-/- mice. Synovial fibroblasts obtained from both mouse groups served as positive controls. Articular chondrocytes were isolated from femoral epiphyseal cartilage as described previously [25]. Murine bone marrow stromal cells were obtained by flushing the bone marrow from femur and tibia with PBS. Cells were maintained in DMEM with 20% FBS and antibiotics until 70% confluence, when they were differentiated into osteoblasts by treatment with 100 μM ascorbate phosphate and 5 mM β-glycerol phosphate for 10 days and medium replacement every 2 days [26]. Other bone marrow cells were differentiated into osteoclasts by their culture in α-MEM (Sigma-Aldrich) supplemented with 10% FBS and antibiotics, 25 ng/ml recombinant macrophage colony stimulating factor and 50 ng/ml RANKL (PreproTech, Rocky Hill, NJ, USA) for 10 days and medium replacement every 2 days [27]. All cells were processed for PCR.

The purity of the different cell types has been documented [27–29] and was confirmed by the mRNA expression of specific markers: collagen type II (chondrocytes), runt related transcription factor 2, alkaline phosphatase, and osteocalcin (osteoblasts), and RANK, calcitonin receptor, and TRAP (osteoclast-like cells). Total RNA was isolated, reverse transcribed, and used for the PCR amplification of a 139-bp fragment from exon 5 of the PRL receptor gene (exon 5 is deleted in *Prlr*-/- mice) in a final reaction mixture (10 μl) containing template, 0.02 U/μl of Phusion DNA Polymerase (Thermo Fisher, Waltham, MA, USA), 2 μl of 5X Phusion Buffer HF (Thermo Fisher), 0.4 mM of deoxyribonucleotide triphosphates (dNTPs) and 0.5 μM of each of the primer pairs for *PRLr* (forward 5′-CAC ATA AAG TGG ATC CGA GGT A-3′; reverse 5′-TGA ATG TCC AGA CTA CAA AAC CA -3′), using *Hprt* as loading control (Additional file 1: Table S1). The amplification in the Eppendorf Mastercycler ep Gradient S equipment (Eppendorf, Hamburg, Germany) included denaturation for 2 minutes at 95 °C, followed by 36 cycles of 15 sec at 95 °C, 15 sec at 56 °C, and 30 sec at 72 °C, followed by one cycle of 5 minutes at 72 °C. PCR products were resolved on a 1.2% agarose gel. The specificity of the reaction was confirmed by the lack of amplification products in samples from *Prlr*-/- mice.

### Western blot

Pulverized ankle joints or synovial fibroblasts were resuspended in lysis buffer (0.1 M Tris-HCl, 0.2 M EGTA, 0.2 M EDTA, 100 mM sodium orthovanadate, 50 mM sodium fluoride, 100 mM sodium acid pyrophosphate, 250 mM sucrose, pH 7.5) and total protein (60 μg), subjected to SDS/PAGE, blotted, and probed overnight with 1:1000 anti-PRL receptor (sc-300; Santa Cruz Biotechnology, Santa Cruz, CA, USA), 1:250 anti-phospho-STAT3 (Tyrosine 705) (9131; Cell Signaling, Beverly, MA, USA), 1:250 anti STAT3 (sc-483; Santa Cruz Biotechnology), or 1:1000 anti-β tubulin (ab6046; Abcam, Cambridge, MA, USA) primary antibodies. Blots were washed in Tris-buffered saline/Tween-20 and detection was performed using goat anti-rabbit conjugated to alkaline phosphatase (1:5000) or to horseradish peroxidase (1:10,000) as secondary antibodies (both from Jackson ImmunoResearch Laboratories Inc., West Grove, PA, USA). Protein densities were quantified with Quantity One software (Bio-Rad, Richmond, CA, USA).

### Statistical analysis

The Sigma Stat 7.0 software (Systat Software, San Jose, CA, USA) was used. Data distribution and equality of variances were determined by the D’Agostino-Pearson test. When the distribution was normal and variances were equal, the *t* test was used to evaluate differences between two groups and one-way analysis of variance (ANOVA) followed by Tukey’s post-hoc test was used to compare differences between more than three groups. In the case of data with a non-parametric distribution, statistical differences between two groups and more than three groups were determined by the Mann-Whitney *U* test and Kruskal-Wallis test followed by Dunn’s post-hoc correction, respectively. The threshold for significance was set at *P* < 0.05.

## Results

### The long PRL receptor isoform increases in the joints and PRL reduces the systemic levels of C-reactive protein and TNFα and the expression of transcription factors and cytokines related to Th17 cells and regulatory T cells in the joints of rats subjected to AIA

**Fig. 1:**
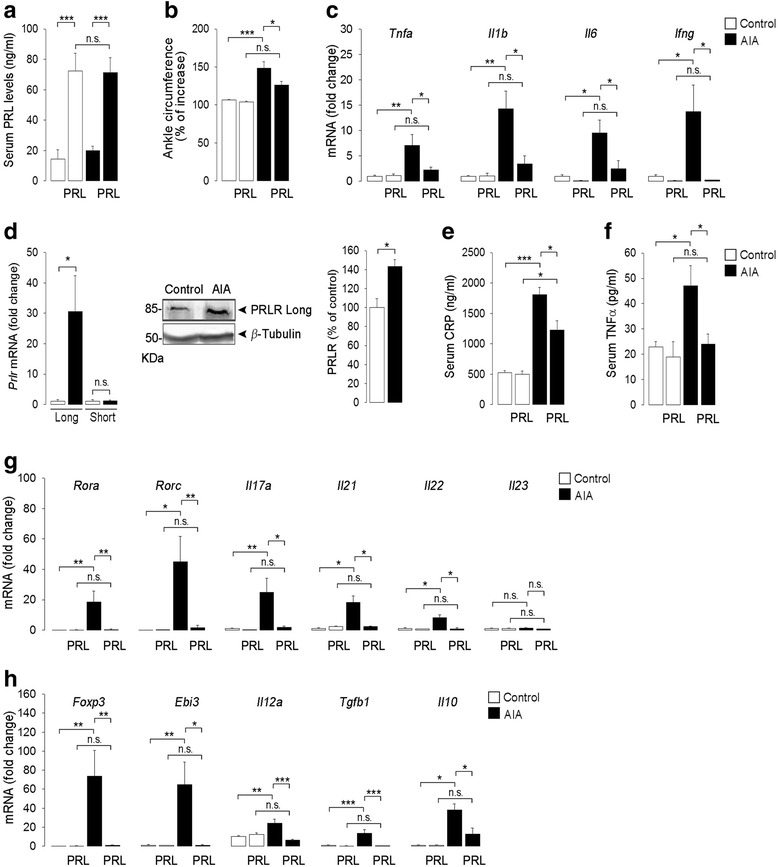
The long prolactin (*PRL*) receptor isoform increases in the joints and PRL reduces the systemic levels of C-reactive protein and TNFα and the expression of transcription factors and cytokines related to T helper 17 (*Th17*) cells and regulatory T cells in the joints of rats subjected to polyarticular adjuvant-induced arthritis (*AIA*)*.* Osmotic minipumps delivering PRL were positioned (or not positioned) 3 days before the intradermal injection of complete Freund’s adjuvant (*CFA*) to induce AIA in rats. All evaluations were performed 21 days after injection of CFA. **a** Serum PRL levels, **b** ankle circumference, and **c** quantitative real-time PCR (qRT-PCR) quantification of *Tnfa*, *Il1b, Il6,* and *Ifng* mRNA levels in ankle joints. **d** qRT-PCR and western blot evaluation of PRL receptor (*Prlr*) long (*Long*) and short (*Short*) isoform mRNA and long PRL receptor protein (*PRLR Long*), respectively. *Bars* show the quantification of PRLR Long/β-Tubulin by densitometry (n = 3). **e** C-reactive protein (*CRP*) and **f** TNFα levels in serum. **g** qRT-PCR quantification of the mRNA levels of the Th17-cell-related transcription factors, *Rora* and *Rorc,* and cytokine genes *Il17a*, *Il21*, *Il22*, and *Il23* in the ankle joints. **h** qRT-PCR quantification of the mRNA levels of the regulatory-T-cell-related transcription factor *Foxp3* and cytokine genes *Ebi3*, *Il12a*, *Tgfb1*, *Il10* in the ankle joints. Values are means ± SEM (n = 5–12). *P < 0.05, **P < 0.01, ***P < 0.001, *n.s.* non-significant

Confirming our previous findings [18], osmotic minipumps delivering PRL, implanted 3 days before the injection of CFA, elevated serum PRL fivefold (Fig. 1a), reduced ankle joint swelling (ankle circumference, Fig. 1b), and lowered joint expression of the proinflammatory cytokine genes, *Tnfa, Il1b, Il6, and Ifng* (Fig. 1c) at day 21 after inducing AIA with CFA in rats. At this time, the levels of the long form of the PRL receptor mRNA and protein increased in the AIA joints, whereas no changes in the transcript of the short PRL receptor isoform were detected (Fig. 1d). PRL infusion in AIA was associated with reduced serum levels of C-reactive protein (CRP*,* Fig. 1e) and TNFα (Fig. 1f), two biomarkers of systemic inflammation in arthritis. Moreover, the imbalance between Th17 and T-regulatory lymphocytes influences inflammation and osteoclastogenesis in arthritis [30], and PRL blocked the AIA-induced increase in the joint expression of the genes *Rorc* and *Rora* encoding for retinoic acid orphan nuclear receptors RORγt and RORα, respectively, which are transcription factors specific for Th17 cells, and the expression of the Th17 cell cytokine genes, *Il17a, Il21*, and *Il22* (Fig. 1g). Also, PRL inhibited the AIA-induced expression of the gene encoding for forkhead box protein 3 (*Foxp3*), a transcription factor specific for regulatory T cells, and the AIA-induced expression of the regulatory-T-cell-related genes encoding for the cytokines IL-35 (a heterodimer composed of IL12a and Ebi3 subunits), TGFβ-1, and IL-10 (Fig. 1h). PRL had no effects in non-arthritic, control animals (Fig. 1a-c and e-g).

### PRL reduces loss of trabecular bone and osteoclast density in AIA

**Fig. 2:**
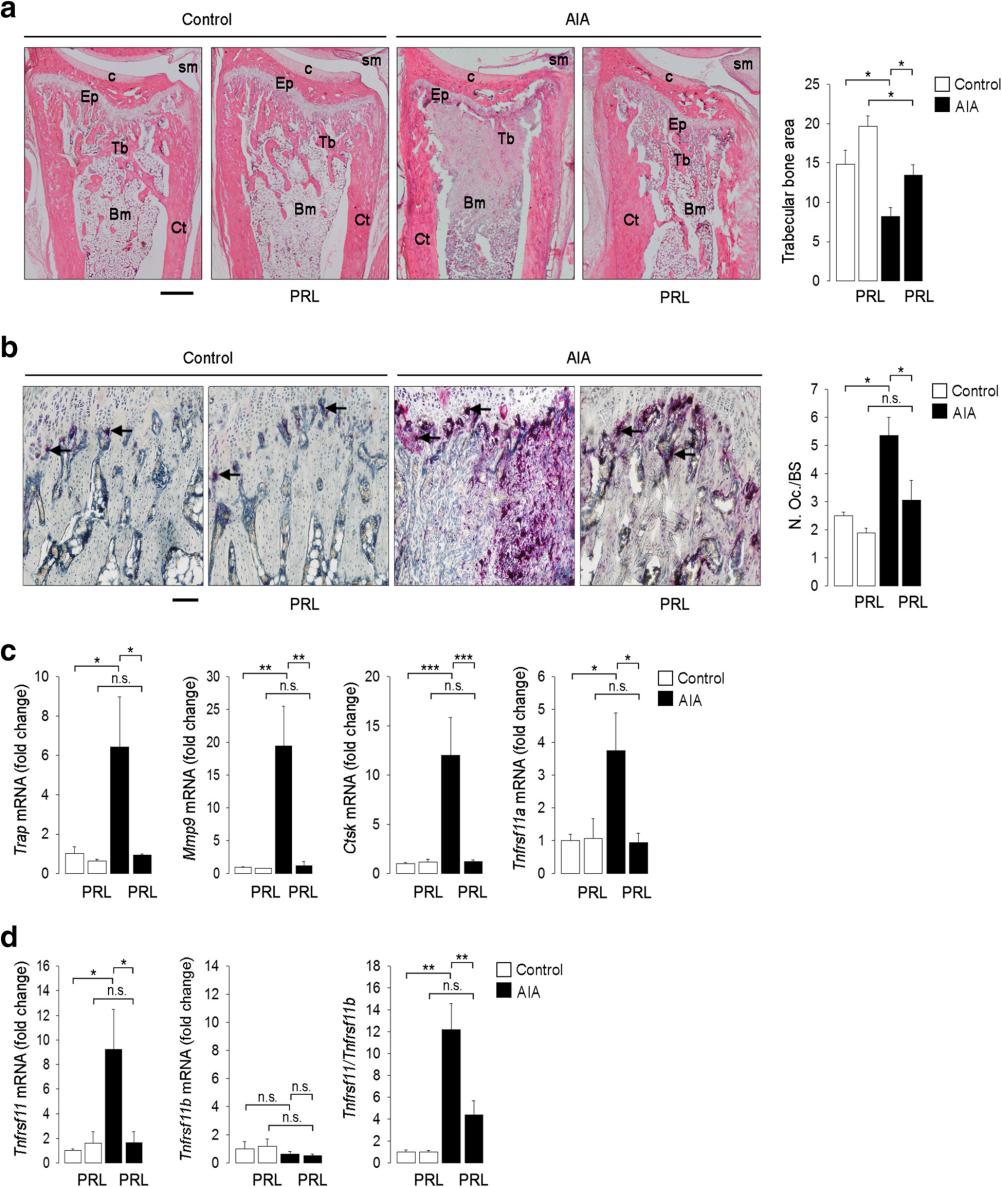
Prolactin (*PRL*) reduces loss of trabecular bone area and osteoclastogenesis in polyarticular adjuvant-induced arthritis (*AIA*). Osmotic minipumps delivering PRL were positioned (or were not positioned) 3 days before the intradermal injection of complete Freund’s adjuvant (*CFA*) to induce AIA in rats. The experiments ended on day 21 after injection of CFA, when all evaluations were performed. **a** Representative images of hematoxylin-eosin-stained tibiotarsal joint sections. *Scale bar* 500 μm. *Ep* epiphyseal plate, *Bm* bone marrow, *Ct* cortical bone, *Tb* trabecular bone, *c* cartilage, *sm* synovial membrane. Graph indicates the values of trabecular bone area (trabecular surface area divided by the total bone area). **b** Representative images of tartrate-resistant alkaline phosphatase (*TRAP*)-stained tibiotarsal joint sections where TRAP-positive *purple spots* (multinucleated cells (osteoclasts)) below the growth plate are indicated (*arrows*). *Scale bar* 200 μm. *Graph* shows the number of osteoclasts per bone surface (*N.Oc*/*BS*). **c** Quantification by quantitative real-time PCR (qRT-PCR) of the mRNA levels of the osteoclast gene markers: *Trap*, *Mmp9*, *Ctsk,* and *Tnfrsf11a* in the ankle joints. **d** qRT-PCR quantification of the mRNA levels of *Tnfrsf11* and *Tnfrsf11b* and of the *Tnfrsf11/Tnfrsf11b* mRNA ratio in the ankle joints. Values are means ± SEM (n = 5–8). *P < 0.05, **P < 0.01, ***P < 0.001, *n.s*. non-significant

Because systemic and local CRP, TNFα, IL-1β, IL-6, and IL-17A trigger osteoclastogenesis by upregulating RANKL in the arthritic joint [2, 31, 32], we evaluated the effect of PRL infusion on trabecular bone loss and osteoclastogenesis in rats with AIA. PRL reduced the loss of trabecular bone area (Fig. 2a) and blocked the increase in osteoclast density in arthritic joints (Fig. 2b). Moreover, PRL decreased osteoclastogenesis as revealed by the reduction in the mRNA levels of the osteoclast-associated genes: *Trap, Mmp9*, *Ctsk*, and the gene encoding for RANK *(Tnfrsf11a)* (Fig. 2c). Also, PRL blocked the AIA-induced increase in the mRNA levels of the gene encoding for RANKL (*Tnfrsf11)* (Fig. 2d). The expression of the gene encoding for OPG (*Tnfrsf11b*) was not modified by AIA or by PRL, but this hormone reduced the *Tnfrsf11/Tnfrsf11b* mRNA ratio in the arthritic joint (Fig. 2d). PRL had no effects on trabecular bone area and osteoclastogenesis in the joints of control rats.

### MAIA-induced joint inflammation and expression of proinflammatory cytokines and that of transcription factors and cytokines related to Th17 cells and regulatory T cells are enhanced in PRL-receptor-null mice

**Fig. 3:**
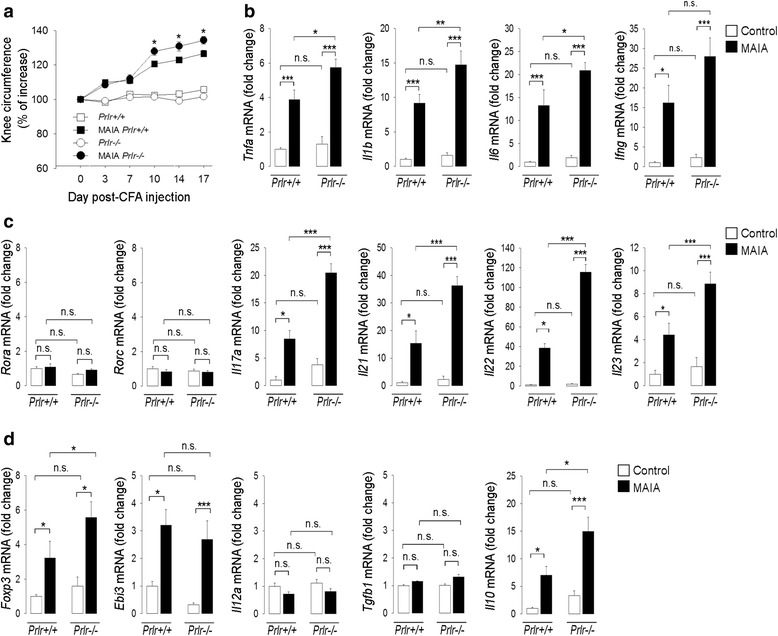
Joint inflammation and expression of proinflammatory cytokines and of transcription factors and cytokines related to T helper 17 (*Th17*) cells and regulatory T cells are enhanced in prolactin (*PRL*)-receptor-null mice with monoarticular adjuvant-induced arthritis (*MAIA*). Mice null (*Prlr*-/-) or not (*Prlr*+/+) for PRL receptors were injected with complete Freund’s adjuvant (*CFA*) into the right knee joint once every 7 days to induce MAIA. The experiments ended on day 18 after the initial CFA injection, when all evaluations were performed. **a** Time course of knee circumference in control and MAIA groups. **b** Quantification by quantitative real-time PCR (*qRT-PCR*) of *Tnfa*, *Il1b, Il6,* and *Ifng* mRNA levels in the knee joints. **c** qRT-PCR quantification of the mRNA levels of the Th17-cell-related transcription factors, *Rora* and *Rorc,* and cytokine genes *Il17a*, *Il21*, *Il22*, and *Il23* in the knee joints. **d** qRT-PCR quantification of the mRNA levels of the regulatory-T-cell-related transcription factor *Foxp3* and cytokine genes *Ebi3*, *Il12a*, *Tgfb1*, *Il10* in the knee joints. Values are means ± SEM (n = 8–12). *P < 0.05, **P < 0.01, ***P < 0.001, *n.s.* non-significant

To further explore the protective effect of PRL against inflammation and osteoclastogenesis in arthritis, mice null (*Prlr*-/-) or not (*Prlr*+/+*)* for the PRL receptor were subjected to MAIA, as this is a highly efficient model for inducing unilateral joint inflammation and bone erosion in the genetic background (C57BL6) of these mice [23]. Inflammation was greater (Fig. 3a) and the expression of the proinflammatory cytokine genes, *Tnfa, Il1b, Il6, Infg* (Fig. 3b), the Th17 cell-related genes, *Il17a, Il21*, *Il22, Il23* (Fig. 3c); and the regulatory-T-cell-associated genes, *Foxp3* and *Il10,* were significantly elevated (Fig. 3d) in the arthritic knee-joint of *Prlr*-/- mice compared to *Prlr*+/+ mice. The expression of *Il12a* was increased in MAIA joints but that of *Rora*, *Rorc*, and *Tgfb1* was not, and these four genes remained unchanged in the absence of PRL receptors (Fig. 3c and d).

### Trabecular bone area is reduced and osteoclast density is increased in PRL receptor-null mice subjected to MAIA

**Fig. 4:**
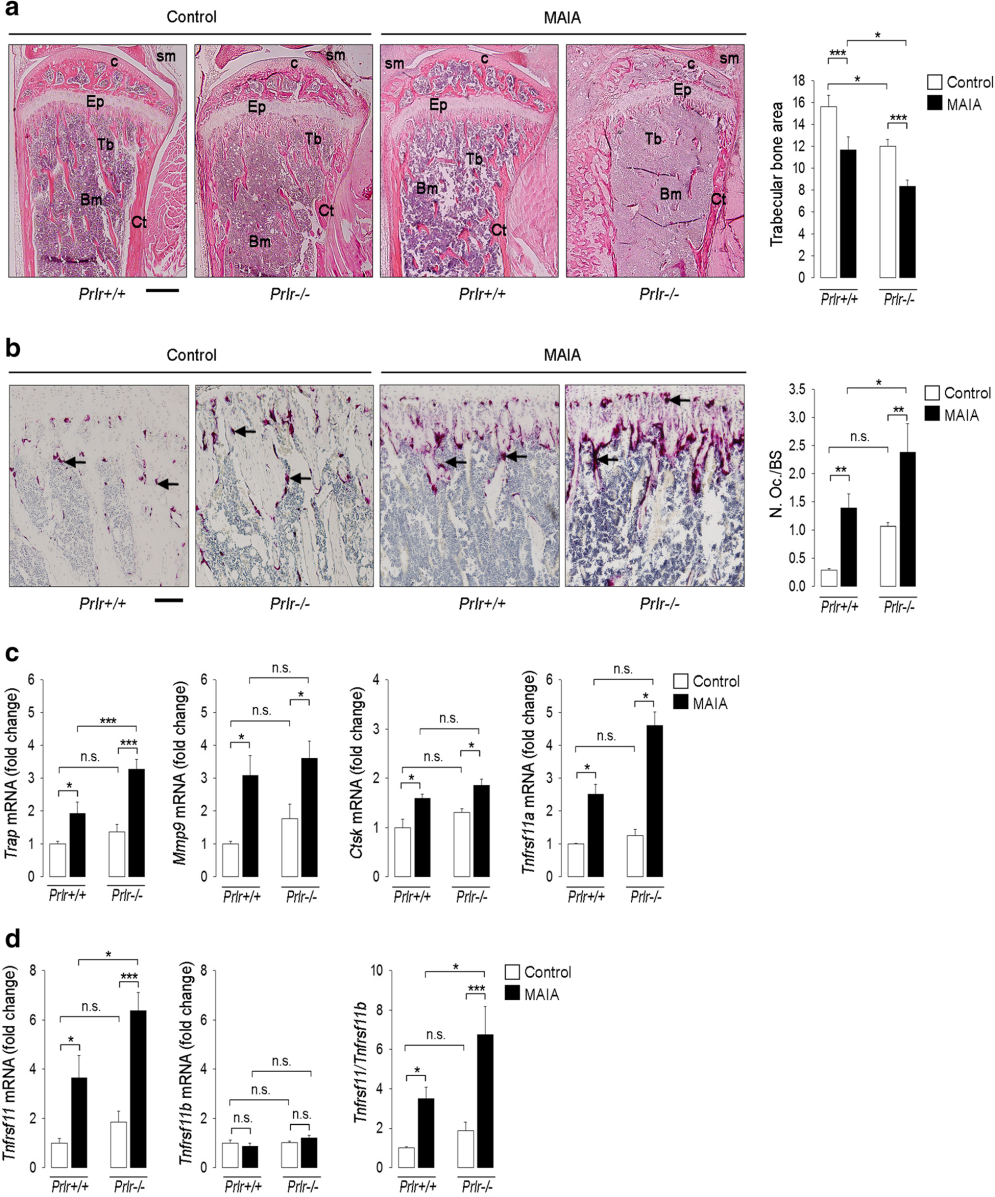
Trabecular bone area is reduced and osteoclastogenesis is enhanced in prolactin (*PRL*)-receptor-null mice subjected to monoarticular adjuvant-induced arthritis (*MAIA*). Mice null (*Prlr*-/-) or not (*Prlr*+/+) for the PRL receptor were injected with complete Freund’s adjuvant (*CFA*) into the right knee joint once every 7 days to induce MAIA. The experiments ended on day 18 after the initial CFA injection, when all evaluations were performed. **a** Representative images of hematoxylin-eosin-stained tibiofemoral joint sections. *Scale bar* 500 μm. *Ep* epiphyseal plate, *Bm* bone marrow, *Ct* cortical bone, *Tb* trabecular bone, *c* cartilage, *sm* synovial membrane. *Graph* indicates the values of trabecular bone area (trabecular surface area divided by the total bone area). **b** Representative images of tartrate-resistance alkaline phosphatase (*TRAP*)-stained tibiofemoral joint sections where TRAP-positive *purple spots* (multinucleated cells (osteoclasts)) below the growth plate are indicated (*arrows*). *Scale bar* 200 μm. *Graph* shows the number of osteoclast per bone surface (*N.Oc./BS*). **c** Quantification by quantitative real-time PCR (*qRT-PCR*) of the mRNA levels of the osteoclast gene markers: *Trap*, *Mmp9*, *Ctsk,* and *Tnfrsf11a* in the knee joints. **d** qRT-PCR-based quantification of the mRNA levels of *Tnfrsf11* and *Tnfrsf11b* and of the *Tnfrsf11/Tnfrsf11b* mRNA ratio in the knee joints. Values are means ± SEM (n = 5–12). *P < 0.05, **P < 0.01, ***P < 0.001, *n.s*. non-significant

Confirming that PRL-receptor-null mice are osteopenic [19], the loss of trabecular bone area was greater in control non-arthritic *Prlr*-/- mice relative to *Prlr*+/+ mice (Fig. 4a). Moreover, the trabecular bone area was reduced (Fig. 4a), whereas the density of osteoclasts (Fig. 4b) and the mRNA levels of *Trap* and *Tnfrsf11* (Fig. 4c, d) were higher in the arthritic joints of *Prlr*-/- mice compared to wild-type mice. There were no differences in the expression levels of *Mmp9*, *Ctsk*, *Tnfrsf11a,* and *Tnfrsf11b* between *Prlr*-/- and *Prlr*+/+ mice, but the *Tnfrsf11/Tnfrsf11b* ratio was higher in *Prlr*-/- mice. Non-arthritic, control *Prlr*-/- mice showed elevated, albeit non-significant, osteoclastogenesis parameters relative to non-arthritic, control *Prlr*+/+ mice (Fig. 4b, c, d). These findings indicate that bone loss and osteoclast development are potentiated in arthritic joints in the absence of PRL signaling and that this hormone influences the maintenance of bone mass under normal conditions.

### TNFα, IL-1β, and IFNγ induce the expression of the long PRL receptor in synovial membranes and synovial fibroblasts

**Fig. 5:**
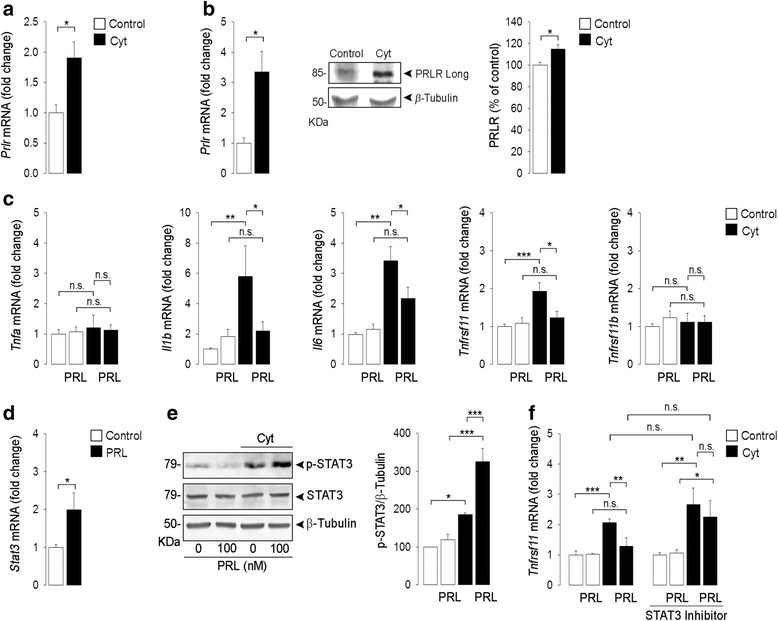
TNFα, IL-1β, and IFNγ (*Cyt*) induce the expression of the long prolactin (*PRL*) receptor (*Prlr*) in synovial membrane and synovial fibroblasts and PRL blocks Cyt-induced *Tnfrsf11* expression in synovial fibroblasts by a STAT3-dependent pathway. **a** Quantification by quantitative real-time PCR (*qRT-PCR*) of PRL receptor long isoform mRNA levels in synovial membrane from mouse knees injected with or without Cyt. **b** qRT-PCR and western blot evaluation of PRL receptor long isoform mRNA and protein in primary cultures of synovial fibroblasts incubated with or without Cyt. *Bars* show the quantification of PRLR Long/β-Tubulin by densitometry. **c** qRT-PCR quantification of *Tnfa*, *Il1b, Il6, Tnfrsf11,* and *Tnfrsf11b* mRNA levels in primary cultures of synovial fibroblasts incubated or not with Cyt in the absence or presence of 100 nM PRL. **d** qRT-PCR and **e** western blot evaluation of *Stat3* mRNA levels and phosphorylated STAT3 (*p-STAT3*), respectively, in primary cultures of synovial fibroblasts incubated with or without Cyt and with or without 100 nM PRL. *Bars* show the quantification of pSTAT3/β-Tubulin by densitometry. **f** qRT-PCR quantification of *Tnfrsf11* mRNA levels in primary cultures of synovial fibroblasts incubated with or without Cyt and with or without 100 nM PRL in the absence or presence of 50 μM of the STAT3 inhibitor S31-201. Values are means ± SEM of three independent experiments. *P < 0.05, **P < 0.01, ***P < 0.001, *n.s.* non-significant

To further investigate the role of PRL receptor signaling in the arthritic joint, a combination of proinflammatory cytokines (Cyt: TNFα, IL-1β, and IFNγ) was injected into the intra-articular space of knee joints of mice in an attempt to mimic the AIA inflammatory condition. The intra-articular Cyt injection elevated the levels of the long form of the PRL receptor mRNA in whole synovial membranes 48 hours after injection (Fig. 5a). Likewise, Cyt treatment for 24 hours stimulated the expression of the long PRL receptor mRNA and protein in primary cultures of mouse synovial fibroblasts (Fig. 5b), implying that these cells become relevant targets of PRL under inflammatory conditions.

### PRL blocks Cyt-induced Tnfrsf11 expression in synovial fibroblasts by a STAT3-dependent pathway

We next studied the contribution of synovial fibroblasts to the anti-osteoclastogenic effects of PRL by culturing synovial fibroblasts with Cyt in the absence or presence of PRL. Cyt induced the expression of the osteoclastogenesis-inducing cytokine genes *Il1b, Il6,* and *Tnfrsf11,* and this effect was blocked by PRL (Fig. 5c). Cyt and PRL did not alter the expression of *Tnfa* or *Tbfrsf11b* (Fig. 5c). Because PRL signals through STAT3 to promote chondrocyte survival [18] and STAT3 inhibits osteoclastogenesis [33], we examined whether PRL signals through STAT3 in synovial fibroblasts. PRL stimulated the expression of *Stat3* mRNA in synovial fibroblasts in culture (Fig. 5d) and promoted the phosphorylation/activation of STAT3 when the cells were cultured in the presence of Cyt (Fig. 5e). Moreover, incubation of synovial fibroblasts with the STAT3 inhibitor S31-201 [34] blocked PRL inhibition of Cyt-induced *Tnfrsf11* expression (Fig. 5f), thereby suggesting that PRL signals through STAT3 to inhibit osteoclastogenesis in joints under inflammatory conditions.

### PRL reduces Cyt-induced osteoclast formation in co-cultures of synovial fibroblasts and osteoclast progenitor cells

**Fig. 6:**
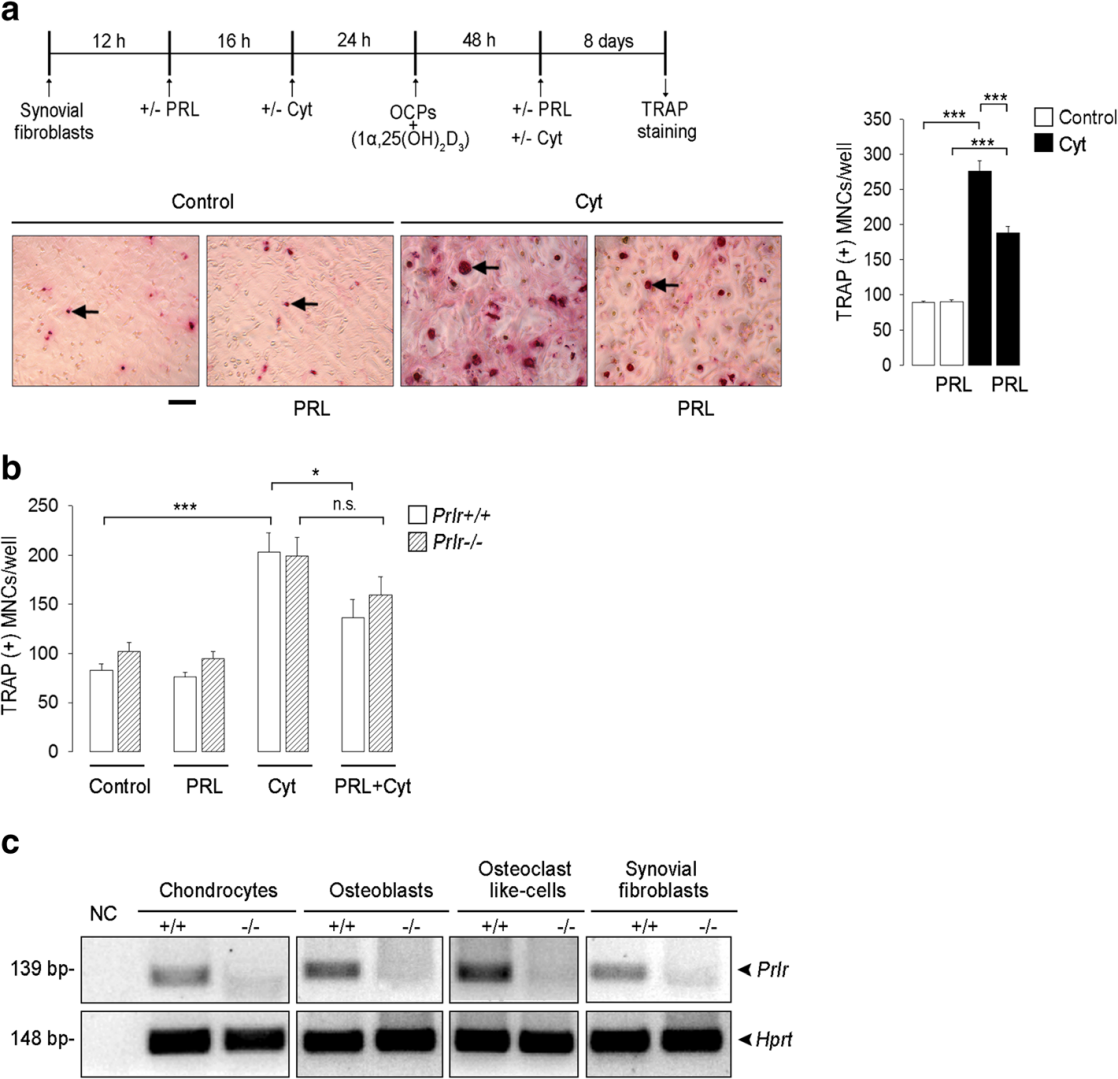
Prolactin (*PRL*) reduces TNFα, IL-1β, and IFNγ (*Cyt*)-induced osteoclast formation in co-cultures of synovial fibroblast and osteoclast progenitor cells. **a** Time course of the co-culture: 12 hours after being seeded, synovial fibroblasts were treated or not with PRL for 16 hours. Cyt were then added or not to the culture and after 24 hours, osteoclast progenitors were delivered in medium containing 1, 25-dihydroxy-vitamin D3. Co-cultures were incubated for 10 days, replacing with new medium (containing +/- Cyt, +/- PRL, and 1, 25-dihydroxy-vitamin D3) every 2 days. Both synovial fibroblasts and osteoclast progenitors were from wild-type (*Prlr*+/+) mice. Osteclastogenesis, was assessed by the number of tartrate-resistant alkaline phosphatase (*TRAP*)-positive, multinucleated (≥3 nuclei) cells (*TRAP*(*+*) *MNCs*) (*arrows*). *Scale bar* 120 μm. **b** Number of TRAP(+) MNCs in co-cultures of synovial fibroblasts from *Prlr*+/+ mice and osteoclast progenitors from *Prlr*+/+ mice or from mice null for the PRL receptor (*Prlr*-/-). Co-cultures were carried out under the same conditions as in **a** Values are means ± SEM (n = 5). *P < 0.05, ***P < 0.001. **c** Real-time PCR amplification of the PRL receptor in joint cells obtained from *Prlr*+/+ and *Prlr*-/- mice. mRNA levels of *Hprt* were amplified as internal control. *NC* without RNA negative control

To investigate synovial fibroblasts and osteoclast progenitor cells as targets of PRL inhibition of osteoclastogenesis, we evaluated the effect of PRL on synovial fibroblasts co-cultured with bone-marrow-derived osteoclast progenitors in the presence of Cyt, which act on synovial fibroblasts to induce the secretion of RANKL necessary for osteoclast differentiation [6] (Fig. 6a). The time course of the co-culture was as follows: 12 hours after being seeded, synovial fibroblasts were treated or not with PRL for 16 hours. Cyt were then added or not to the culture and after 24 hours osteoclast progenitors were delivered in medium containing 1, 25-dihydroxy-vitamin D3. Co-cultures were incubated for 10 days, replacing with new medium (containing +/- Cyt, +/- PRL, and 1, 25-dihydroxy-vitamin D3) every 2 days. Cyt treatment promoted the differentiation of osteoclast precursors into TRAP+, enlarged, multinucleated cells (mature osteoclasts), and this effect was significantly reduced by PRL (Fig. 6a). PRL can act on synovial fibroblasts to inhibit osteoclastogenesis (Fig. 5). To investigate osteoclast progenitors as PRL targets, synovial fibroblasts derived from *Prlr*+/+ mice were co-cultured with osteoclast progenitors from either *Prlr*+/+ mice or *Prlr*-/- mice. PRL had no significant effect on the number of mature osteoclasts when osteoclast precursors were derived from *Prlr*-/- mice. However, the number of mature osteoclasts was similar between the co-cultures treated with Cyt and PRL, whether or not osteoclast progenitors were derived from PRL-receptor-null mice (Fig. 6b). These findings support a direct effect of PRL on both synovial fibroblasts and osteoclast progenitors to reduce osteoclastogenesis.

### Expression of PRL receptor transcripts in chondrocytes, osteoblasts, and osteoclast-like cells

In addition to synovial fibroblasts, other cells in joint tissues express PRL receptors. RT-PCR performed on chondrocytes, osteoblasts, osteoclast-like cells, and synovial fibroblasts (positive controls) from *Prlr*+/+ mice revealed a single band of 139 bp, the size of which corresponded to the region amplified from exon 5 of the *Prlr* gene (Fig. 6c). Specificity of the reaction was confirmed by the absence of such a transcript in cells obtained from *Prlr*-/- mice. In these knockout mice, exon 5 of the PRL receptor has been deleted, which results in a very short peptide unable to bind to PRL.

## Discussion

Articular bone loss and increased fracture risk in arthritis are indicative of an imbalance between bone resorption and formation. Bone resorption depends on the development of bone resorbing osteoclasts (osteoclastogenesis) in response to systemic and local signals that converge to regulate the RANKL/RANK/OPG system [2, 3, 7, 8]. Here, we showed that PRL reduces the systemic levels and the joint production of cytokines with osteoclastogenic activity, lowers the joint expression of the genes encoding for RANKL and RANK, decreases osteoclast density, and reduces trabecular bone area in two murine models of inflammatory arthritis. Moreover, synovial fibroblasts are an important source for RANKL-induced bone loss in arthritis [6], and we showed that PRL downregulates cytokine-induced expression of the RANKL gene in cultured synovial fibroblasts via a STAT3-dependent pathway, and that this hormone reduces osteoclastogenesis in co-cultures of synovial fibroblasts and osteoclast progenitor cells.

Chronic inflammation determines local and systemic bone loss in RA [2, 3] and may be attenuated by PRL. PRL treatment reduces joint swelling, proinflammatory cytokine expression (TNFα, IL-1β, IL-6, and IFNγ), pannus formation, and the destruction of bone trabeculae in rats with AIA [18], a model of inflammatory arthritis. Here, we have extended these findings by showing that increasing serum PRL to levels similar to those (40 ng/ml) found in the circulation of some patients with RA [35] reduced systemic levels of CRP and TNFα, two osteoclastogenic cytokines [2, 3, 31]. The serum levels of CRP and TNFα in RA patients correlate with their levels in the synovial fluid and reflect systemic and joint inflammatory responses [31, 36]. Moreover, PRL treatment reduced the expression of transcription factors and cytokines associated with Th17 and T regulatory cells in arthritic joints. Th17 cells promote inflammation, osteoclastogenesis, and bone erosion in arthritis [32, 37], whereas T regulatory cells suppress immune responses [38], and the imbalance of the two T lymphocyte subpopulations has been identified as a key event in the pathogenesis of rheumatoid arthritis [30]. It remains to be determined how PRL actions, particularly the downregulation of T regulatory cells, would contribute to PRL protection against inflammation and osteoclastogenesis in arthritis. The matter is complex as the differentiation and function of Th17 and T regulatory cells are closely related and vary in the presence of strong inflammatory conditions [39, 40]. Both T cell populations require transforming growth factor (TGF)β for differentiation [41], and in vivo studies have identified a subset of T cells that dually express elements of both the T regulatory and Th17 phenotypes [40, 42] with potent inflammatory and osteoclastogenic effects in autoimmune arthritis [43].

In support of PRL acting locally on inflamed joint tissues, we show that the long PRL receptor isoform is upregulated in the joints of rats with AIA and in the synovial membranes and synovial fibroblasts of mice treated with the proinflammatory cytokines, TNFα, IL-1β, and IFNγ, which contribute to AIA inflammatory lesions [44]. PRL receptors exist in various molecular forms that differ primarily in the sequence and length of their cytoplasmic domain and are classified as long, intermediate, and short [45]. The long PRL receptor is considered the major isoform signaling all PRL actions, the intermediate isoform transmits cell proliferation and survival signals, and the short PRL receptor exerts dominant-negative effects on signals by the long form [46]. The long form of the PRL receptor appears to predominate in osteoblasts [19] and chondrocytes [25], where PRL promotes bone development and cartilage survival. Also, fibroblasts and T cells in the synovium of patients with RA express PRL receptors [47], and proinflammatory cytokines induce the expression of the long form of the PRL receptor in lung fibroblasts to mediate anti-inflammatory effects of PRL in the airways [48]. Therefore, these findings could imply that the long form of the PRL receptor mediates the protective effects of PRL in arthritis.

Supporting the anti-inflammatory role of PRL in arthritis, PRL-receptor-null mice exhibited increased joint swelling and higher joint expression of the genes encoding for TNFα, IL-1β, IL-6, IFNγ, IL-17A, IL-21, IL-22, IL-23, and IL-10 when subjected to monoarticular AIA (MAIA). These findings are consistent with PRL treatment inhibiting the expression of the same cytokines in the joints of rats with AIA [18] and with previous reports showing that targeted disruption of PRL [13] and of PRL receptors [49] enhances immune responses and mortality under stress-related conditions. Besides their crucial effects on joint inflammation, TNFα, IL-1β, IL-6, and IL-17A also drive bone loss. They promote osteoclastic bone resorption largely by stimulating the expression and function of RANKL and RANK [2, 32]. Accordingly, we next investigated whether PRL downregulates osteoclast development and bone loss in AIA and MAIA.

Increasing systemic PRL levels in rats with AIA and blocking PRL receptor signaling in *Prlr*-/- mice with MAIA reduced and increased, respectively, the loss of trabecular bone area, osteoclast density, the accumulation of phenotypic markers identifying osteoclasts (the gene for RANKL cognate receptor RANK and the genes encoding for acid (TRAP and cathepsin K) and neutral (MMP-9) proteases used by osteoclasts to degrade the organic matrix of bone [3]). Moreover, PRL and lack of PRL signaling reduced and increased, respectively, the expression of RANKL gene (*Tnfrsf11*) in the arthritic joint, which resulted in a lower and higher *Tnfrsf11/Tnfrsf11b* mRNA ratio for each condition, respectively. *Tnfrsf11b* encodes for OPG, a soluble decoy receptor that neutralizes RANKL and prevents bone erosion in AIA [4]. A high RANKL/OPG ratio occurs in patients with RA and is associated with increased bone resorption [50].

Our findings indicate that inhibition of RANKL-induced osteoclast development is a component of PRL protection against bone loss in inflammatory arthritis. It is unclear whether PRL signals directly on osteoclasts or indirectly through other cell types responsible for bone loss. Arguing against osteoclasts being direct targets of PRL is the finding that bone marrow cells differentiated into osteoclasts are devoid of the PRL receptor [19]. On the other hand, chondrocytes, osteoblasts, and synovial fibroblasts express PRL receptors and are major sources of osteoclastogenic cytokines including RANKL [2, 5, 6, 51]. Here, we support direct effects of PRL on chondrocytes, synovial fibroblasts, and osteoblasts, but also in osteoclasts by showing the PCR-mediated amplification of a PRL receptor transcript in the various cell types obtained from *Prlr*+/+ mice but not in those obtained from *Prlr*-/- mice. While differences in osteoclast differentiation protocols may contribute to our contrasting finding [19], the presence of the PRL receptor in osteoclasts reinforces the role of PRL on bone remodeling.

Consistent with synovial fibroblasts being cellular targets of PRL-induced anti-osteoclastogenesis, the incubation of primary cultures of synovial fibroblasts with TNFα, IL-1β, and IFNγ (Cyt) elevated the expression of the PRL receptor, and treatment of these cells with PRL inhibited Cyt-induced expression of *Il1b*, *Il6*, and *Tnfrsf11*. PRL receptors signal through Janus kinase-2 (JAK-2)-STATs 1, 5, and 3 as their canonical pathway [52]. STATs, and in particular STAT3, are emerging as important regulators of bone homeostasis [33]. STAT3 mutations correlate with increased osteoclast number and bone resorption in the clinic [53, 54] and mice with an osteoblast-specific deletion of *Stat3* have an osteopenic phenotype [33]. Here, we demonstrate that PRL stimulates the expression of *Stat3* and enhances Cyt-induced phosphorylation/activation of STAT3 in synovial fibroblasts. The Cyt-induced STAT3 activation may be due to the production of IL-6 in response to the Cyt, as IL-6 activates STAT3 in synovial fibroblasts [55]. However, IL-6-mediated STAT3 activation may lead to either bone resorption [56] or bone formation [57]. In support of STAT3 being anti-osteoclastogenic when activated by PRL, pharmacological blockage of STAT3 prevented PRL inhibition of Cyt-induced *Tnfrsf11* expression. These findings suggest that PRL may signal through STAT3 in synovial fibroblasts to inhibit RANKL-induced osteoclastogenesis. Consistent with this notion, we found that PRL acts on co-cultures of synovial fibroblasts and osteoclast precursor cells to downregulate Cyt-induced osteoclast differentiation. The fact that such PRL inhibition was reduced when osteoclast precursors were derived from *Prlr*-/- mice implies that PRL receptors in osteoclast progenitor cells contribute to PRL protection against osteoclastogenesis. This is in agreement with the presence of PRL receptors in osteoclast-like cells and with the PRL downregulation of RANK in arthritic joints. Reduction of RANK levels may contribute to PRL inhibitory effects on osteoclastogenesis and bone loss at the level of osteoclast progenitors and mature osteoclasts.

PRL regulation of the RANKL/RANK/OPG system for physiological bone remodeling during growth and reproduction is well-substantiated [58]. PRL decreases the RANKL/OPG ratio by downregulating RANKL and upregulating OPG in osteoblasts to promote bone formation early in life [20], whereas in pregnancy and lactation, PRL accelerates bone turnover by raising the RANKL/OPG ratio in osteoblasts [59, 60] in order to supply calcium for fetal growth and milk production. The opposing effects of PRL on bone remodeling indicate that the outcome of its action depends on complex age-related and hormone-related interactions.

In experimental inflammatory arthritis, PRL appears to be beneficial; however, the role of PRL in RA is controversial (as reviewed [58]). PRL increases in the circulation of some patients with RA, but it is not clear whether systemic PRL levels correlate with disease severity. Confounding factors include the contribution of PRL synthesized locally by joint tissues like chondrocytes [61], endothelial cells [62], synoviocytes and immune cells [47]; and the ability of PRL to exert immunostimulatory or immunosuppressive effects, depending on its level and that of other cytokines and hormones [58]. Hyperprolactinemia in the context of reproduction [59] and non-inflammatory pathology (prolactinoma) [63] promotes bone loss, whereas high circulating PRL levels can be anti-inflammatory and reduce bone loss under inflammatory conditions [18, 64] (and present findings). The mechanisms governing the effects of PRL on bone remodeling are challenging but a better understanding of them could lead to the use of hyperprolactinemia-inducing drugs as therapeutic agents in the clinic.

## Conclusion

We demonstrated in this study that PRL protects against bone loss and osteoclastogenesis in inflammatory arthritis by inhibiting cytokine-induced activation of RANKL in joints and synovial fibroblasts via its canonical STAT3 signaling pathway.

## Additional file

Additional file 1: Table S1. Primers used for qRT-PCR. Primers and their annealing temperatures used in quantitative RT-PCR studies. (DOCX 24 kb)

## Abbreviations

AIA: Adjuvant-induced arthritis
bp: Base pairs
CD90.2: Cluster of differentiation 90
CFA: Complete Freund’s adjuvant
CRP: C-reactive protein
*Ctsk*: Cathepsin K gene
Cyt: Combination of the proinflammatory cytokines TNFα, IL-1β, and interferon-γ
DMEM: Dulbecco’s modified Eagle’s medium
*Ebi3*: Gene encoding for Epstein-Barr virus induced 3
ELISA: Enzyme-linked immunosorbent assay
FBS: Fetal bovine serum
*Foxp3*: Gene encoding for forkhead box protein 3
Hprt: Hypoxanthine-guanine phosphoribosyltransferase
ICAM-1: Intercellular adhesion molecule 1
IFNγ: Interferon γ
IL: Interleukin
*Il10*: Gene encoding for interleukin 10
*Il12a*: Gene encoding for interleukin 12A
JAK-2: Janus kinase-2
MAIA: Monoarticular adjuvant-induced arthritis
Mmp9: Matrix metalloproteinase 9
OPG: Osteoprotegerin
PBS: phosphate-buffered saline
PRL: Prolactin
PRLR: Prolactin receptor
qPCR: Quantitative polymerase chain reaction
qRT-PCR: Quantitative real-time polymerase chain reaction
RA: Rheumatoid arthritis
RANK: Receptor activator of nuclear factor κB
RANKL: Receptor activator of nuclear factor κB ligand
*Rora*: Gene encoding for retinoic acid orphan nuclear receptors RORα
*Rorc*: Gene encoding for retinoic acid orphan nuclear receptors RORγt
STAT3: Signal transducer and activator of transcription 3
*Tgfb1*: Gene encoding for transforming growth factor β 1
Th: T helper
*Tnfrsf11*: Gene encoding for RANKL
*Tnfrsf11a*: Gene encoding for RANK
*Tnfrsf11b*: Gene encoding for OPG
TNFα: Tumor necrosis factor alpha
TRAP: Tartrate-resistant acid phosphatase
VCAM1: Vascular cell adhesion molecule 1

## References

[1] Cohen SB, Dore RK, Lane NE, Ory PA, Peterfy CG, Sharp JT, van der Heijde D, Zhou L, Tsuji W, Newmark R (2008). Denosumab treatment effects on structural damage, bone mineral density, and bone turnover in rheumatoid arthritis: a twelve-month, multicenter, randomized, double-blind, placebo-controlled, phase II clinical trial. Arthritis Rheum.

[2] Braun T, Zwerina J (2011). Positive regulators of osteoclastogenesis and bone resorption in rheumatoid arthritis. Arthritis Res Ther.

[3] Schett G, Gravallese E (2012). Bone erosion in rheumatoid arthritis: mechanisms, diagnosis and treatment. Nat Rev Rheumatol.

[4] Kong YY, Feige U, Sarosi I, Bolon B, Tafuri A, Morony S, Capparelli C, Li J, Elliott R, McCabe S (1999). Activated T cells regulate bone loss and joint destruction in adjuvant arthritis through osteoprotegerin ligand. Nature.

[5] Gravallese EM, Manning C, Tsay A, Naito A, Pan C, Amento E, Goldring SR (2000). Synovial tissue in rheumatoid arthritis is a source of osteoclast differentiation factor. Arthritis Rheum.

[6] Tunyogi-Csapo M, Kis-Toth K, Radacs M, Farkas B, Jacobs JJ, Finnegan A, Mikecz K, Glant TT (2008). Cytokine-controlled RANKL and osteoprotegerin expression by human and mouse synovial fibroblasts: fibroblast-mediated pathologic bone resorption. Arthritis Rheum.

[7] Hofbauer LC, Heufelder AE (2001). Role of receptor activator of nuclear factor-kappaB ligand and osteoprotegerin in bone cell biology. J Mol Med (Berl).

[8] Jones DH, Kong YY, Penninger JM (2002). Role of RANKL and RANK in bone loss and arthritis. Ann Rheum Dis.

[9] Brennan P, Ollier B, Worthington J, Hajeer A, Silman A (1996). Are both genetic and reproductive associations with rheumatoid arthritis linked to prolactin?. Lancet.

[10] Neidhart M, Gay RE, Gay S (1999). Prolactin and prolactin-like polypeptides in rheumatoid arthritis. Biomed Pharmacother.

[11] Costanza M, Binart N, Steinman L, Pedotti R (2015). Prolactin: a versatile regulator of inflammation and autoimmune pathology. Autoimmun Rev.

[12] Matera L, Cesano A, Bellone G, Oberholtzer E (1992). Modulatory effect of prolactin on the resting and mitogen-induced activity of T, B, and NK lymphocytes. Brain Behav Immun.

[13] Dugan AL, Thellin O, Buckley DJ, Buckley AR, Ogle CK, Horseman ND (2002). Effects of prolactin deficiency on myelopoiesis and splenic T lymphocyte proliferation in thermally injured mice. Endocrinology.

[14] Oberbeck R, Schmitz D, Wilsenack K, Schuler M, Biskup C, Schedlowski M, Nast-Kolb D, Exton MS (2003). Prolactin modulates survival and cellular immune functions in septic mice. J Surg Res.

[15] de Man YA, Dolhain RJ, van de Geijn FE, Willemsen SP, Hazes JM (2008). Disease activity of rheumatoid arthritis during pregnancy: results from a nationwide prospective study. Arthritis Rheum.

[16] Pikwer M, Bergstrom U, Nilsson JA, Jacobsson L, Berglund G, Turesson C (2009). Breast feeding, but not use of oral contraceptives, is associated with a reduced risk of rheumatoid arthritis. Ann Rheum Dis.

[17] Grimaldi MG (1981). Long-term low dose haloperidol treatment in rheumatoid patients: effects on serum sulphydryl levels, technetium index, ESR, and clinical response. Br J Clin Pharmacol.

[18] Adan N, Guzman-Morales J, Ledesma-Colunga MG, Perales-Canales SI, Quintanar-Stephano A, Lopez-Barrera F, Mendez I, Moreno-Carranza B, Triebel J, Binart N (2013). Prolactin promotes cartilage survival and attenuates inflammation in inflammatory arthritis. J Clin Invest.

[19] Clement-Lacroix P, Ormandy C, Lepescheux L, Ammann P, Damotte D, Goffin V, Bouchard B, Amling M, Gaillard-Kelly M, Binart N (1999). Osteoblasts are a new target for prolactin: analysis of bone formation in prolactin receptor knockout mice. Endocrinology.

[20] Seriwatanachai D, Charoenphandhu N, Suthiphongchai T, Krishnamra N (2008). Prolactin decreases the expression ratio of receptor activator of nuclear factor kappaB ligand/osteoprotegerin in human fetal osteoblast cells. Cell Biol Int.

[21] Kondegowda NG, Fenutria R, Pollack IR, Orthofer M, Garcia-Ocana A, Penninger JM, Vasavada RC (2015). Osteoprotegerin and denosumab stimulate human beta cell proliferation through inhibition of the receptor activator of NF-kappaB ligand pathway. Cell Metab.

[22] Srivastava S, Matsuda M, Hou Z, Bailey JP, Kitazawa R, Herbst MP, Horseman ND (2003). Receptor activator of NF-kappaB ligand induction via Jak2 and Stat5a in mammary epithelial cells. J Biol Chem.

[23] Gauldie SD, McQueen DS, Clarke CJ, Chessell IP (2004). A robust model of adjuvant-induced chronic unilateral arthritis in two mouse strains. J Neurosci Methods.

[24] Armaka M, Gkretsi V, Kontoyiannis D, Kollias G (2009). A standardized protocol for the isolation and culture of normal and arthritogenic murine synovial fibroblasts. Protocol Exch.

[25] Zermeno C, Guzman-Morales J, Macotela Y, Nava G, Lopez-Barrera F, Kouri JB, Lavalle C, de la Escalera GM, Clapp C (2006). Prolactin inhibits the apoptosis of chondrocytes induced by serum starvation. J Endocrinol.

[26] Rauner M, Foger-Samwald U, Kurz MF, Brunner-Kubath C, Schamall D, Kapfenberger A, Varga P, Kudlacek S, Wutzl A, Hoger H (2014). Cathepsin S controls adipocytic and osteoblastic differentiation, bone turnover, and bone microarchitecture. Bone..

[27] Marino S, Logan JG, Mellis D, Capulli M (2014). Generation and culture of osteoclasts. Bonekey Rep..

[28] Shakibaei M, De Souza P, Merker HJ (1997). Integrin expression and collagen type II implicated in maintenance of chondrocyte shape in monolayer culture: an immunomorphological study. Cell Biol Int.

[29] Rauner M, Winzer M, Stupphann D, Krenbek D, Pietschmann P (2007). RANKL and OPG gene expression bone marrow stromal cells and calvarial osteoblasts in mouse and rat. Osteologie..

[30] Alunno A, Manetti M, Caterbi S, Ibba-Manneschi L, Bistoni O, Bartoloni E, Valentini V, Terenzi R, Gerli R (2015). Altered immunoregulation in rheumatoid arthritis: the role of regulatory T cells and proinflammatory Th17 cells and therapeutic implications. Mediat Inflamm..

[31] Kim KW, Kim BM, Moon HW, Lee SH, Kim HR (2015). Role of C-reactive protein in osteoclastogenesis in rheumatoid arthritis. Arthritis Res Ther..

[32] Sato K, Suematsu A, Okamoto K, Yamaguchi A, Morishita Y, Kadono Y, Tanaka S, Kodama T, Akira S, Iwakura Y (2006). Th17 functions as an osteoclastogenic helper T cell subset that links T cell activation and bone destruction. J Exp Med.

[33] Li J (2013). JAK-STAT and bone metabolism. Jak-Stat.

[34] Siddiquee K, Zhang S, Guida WC, Blaskovich MA, Greedy B, Lawrence HR, Yip ML, Jove R, McLaughlin MM, Lawrence NJ (2007). Selective chemical probe inhibitor of Stat3, identified through structure-based virtual screening, induces antitumor activity. Proc Natl Acad Sci USA.

[35] Ghule S, Dhotre A, Gupta M, Dharme P, Vaidya S (2009). Serum prolactin levels in women with rheumatoid arthritis. Biomed Res.

[36] Hamed EA, Hamed EA, Hamed SA, Gamal H-A (2007). Synovial fluid and serum levels of sE-selectin, IL-1β and TNF-α in rheumatoid arthritis. JKAU Med Sci.

[37] Lubberts E (2010). Th17 cytokines and arthritis. Semin Immunopathol.

[38] Sakaguchi S, Miyara M, Costantino CM, Hafler DA (2010). FOXP3+ regulatory T cells in the human immune system. Nat Rev Immunol.

[39] Deknuydt F, Bioley G, Valmori D, Ayyoub M (2009). IL-1beta and IL-2 convert human Treg into T(H)17 cells. Clin Immunol.

[40] Diller ML, Kudchadkar RR, Delman KA, Lawson DH, Ford ML (2016). Balancing inflammation: the link between Th17 and regulatory T cells. Mediat Inflamm..

[41] Zhou L, Lopes JE, Chong MM, Ivanov II, Min R, Victora GD, Shen Y, Du J, Rubtsov YP, Rudensky AY (2008). TGF-beta-induced Foxp3 inhibits T(H)17 cell differentiation by antagonizing RORgammat function. Nature.

[42] Voo KS, Wang YH, Santori FR, Boggiano C, Wang YH, Arima K, Bover L, Hanabuchi S, Khalili J, Marinova E (2009). Identification of IL-17-producing FOXP3+ regulatory T cells in humans. Proc Natl Acad Sci USA.

[43] Komatsu N, Okamoto K, Sawa S, Nakashima T, Oh-hora M, Kodama T, Tanaka S, Bluestone JA, Takayanagi H (2014). Pathogenic conversion of Foxp3+ T cells into TH17 cells in autoimmune arthritis. Nat Med.

[44] Panayi GS, Lanchbury JS, Kingsley GH (1992). The importance of the T cell in initiating and maintaining the chronic synovitis of rheumatoid arthritis. Arthritis Rheum.

[45] Bole-Feysot C, Goffin V, Edery M, Binart N, Kelly PA (1998). Prolactin (PRL) and its receptor: actions, signal transduction pathways and phenotypes observed in PRL receptor knockout mice. Endocr Rev.

[46] Ben-Jonathan N, LaPensee CR, LaPensee EW (2008). What can we learn from rodents about prolactin in humans?. Endocr Rev.

[47] Nagafuchi H, Suzuki N, Kaneko A, Asai T, Sakane T (1999). Prolactin locally produced by synovium infiltrating T lymphocytes induces excessive synovial cell functions in patients with rheumatoid arthritis. J Rheumatol.

[48] Corbacho AM, Macotela Y, Nava G, Eiserich JP, Cross CE, Martinez de la Escalera G, Clapp C (2003). Cytokine induction of prolactin receptors mediates prolactin inhibition of nitric oxide synthesis in pulmonary fibroblasts. FEBS Lett.

[49] Moreno-Carranza B, Goya-Arce M, Vega C, Adan N, Triebel J, Lopez-Barrera F, Quintanar-Stephano A, Binart N, Martinez de la Escalera G, Clapp C (2013). Prolactin promotes normal liver growth, survival, and regeneration in rodents: effects on hepatic IL-6, suppressor of cytokine signaling-3, and angiogenesis. Am J Physiol Regul Integr Comp Physiol.

[50] Fadda S, Hamdy A, Abulkhair E, Mahmoud Elsify H, Mostafa A (2015). Serum levels of osteoprotegerin and RANKL in patients with rheumatoid arthritis and their relation to bone mineral density and disease activity. Egyptian Rheumatologist..

[51] Martinez-Calatrava MJ, Prieto-Potin I, Roman-Blas JA, Tardio L, Largo R, Herrero-Beaumont G (2012). RANKL synthesized by articular chondrocytes contributes to juxta-articular bone loss in chronic arthritis. Arthritis Res Ther.

[52] DaSilva L, Rui H, Erwin RA, Howard OM, Kirken RA, Malabarba MG, Hackett RH, Larner AC, Farrar WL (1996). Prolactin recruits STAT1, STAT3 and STAT5 independent of conserved receptor tyrosines TYR402, TYR479, TYR515 and TYR580. Mol Cell Endocrinol.

[53] Holland SM, DeLeo FR, Elloumi HZ, Hsu AP, Uzel G, Brodsky N, Freeman AF, Demidowich A, Davis J, Turner ML (2007). STAT3 mutations in the hyper-IgE syndrome. N Engl J Med.

[54] Minegishi Y, Saito M, Tsuchiya S, Tsuge I, Takada H, Hara T, Kawamura N, Ariga T, Pasic S, Stojkovic O (2007). Dominant-negative mutations in the DNA-binding domain of STAT3 cause hyper-IgE syndrome. Nature.

[55] Hashizume M, Hayakawa N, Mihara M (2008). IL-6 trans-signalling directly induces RANKL on fibroblast-like synovial cells and is involved in RANKL induction by TNF-alpha and IL-17. Rheumatology.

[56] O’Brien CA, Gubrij I, Lin SC, Saylors RL, Manolagas SC (1999). STAT3 activation in stromal/osteoblastic cells is required for induction of the receptor activator of NF-kappaB ligand and stimulation of osteoclastogenesis by gp130-utilizing cytokines or interleukin-1 but not 1,25-dihydroxyvitamin D3 or parathyroid hormone. J Biol Chem.

[57] Shin HI, Divieti P, Sims NA, Kobayashi T, Miao D, Karaplis AC, Baron R, Bringhurst R, Kronenberg HM (2004). Gp130-mediated signaling is necessary for normal osteoblastic function in vivo and in vitro. Endocrinology.

[58] Clapp C, Adan N, Ledesma-Colunga MG, Solís-Gutiérrez M, Triebel J, Martinez de la Escalera G. The role of the prolactin/vasoinhibin axis in rheumatoid arthritis: an integrative overview. Cell Mol Life Sci. 2016;73(15):2929–48. 10.1007/s00018-016-2187-0 PMC11108309 27026299

[59] Lotinun S, Limlomwongse LC, Sirikulchayanonta V, Krishnamra N (2003). Bone calcium turnover, formation, and resorption in bromocriptine- and prolactin-treated lactating rats. Endocrine.

[60] Seriwatanachai D, Thongchote K, Charoenphandhu N, Pandaranandaka J, Tudpor K, Teerapornpuntakit J, Suthiphongchai T, Krishnamra N (2008). Prolactin directly enhances bone turnover by raising osteoblast-expressed receptor activator of nuclear factor kappaB ligand/osteoprotegerin ratio. Bone.

[61] Macotela Y, Aguilar MB, Guzman-Morales J, Rivera JC, Zermeno C, Lopez-Barrera F, Nava G, Lavalle C, Martinez de la Escalera G, Clapp C (2006). Matrix metalloproteases from chondrocytes generate an antiangiogenic 16 kDa prolactin. J Cell Sci.

[62] Corbacho AM, Macotela Y, Nava G, Torner L, Duenas Z, Noris G, Morales MA, Martinez De La Escalera G, Clapp C (2000). Human umbilical vein endothelial cells express multiple prolactin isoforms. J Endocrinol.

[63] Mazziotti G, Porcelli T, Mormando M, De Menis E, Bianchi A, Mejia C, Mancini T, De Marinis L, Giustina A (2011). Vertebral fractures in males with prolactinoma. Endocrine.

[64] Jacobi AM, Rohde W, Volk HD, Dorner T, Burmester GR, Hiepe F (2001). Prolactin enhances the in vitro production of IgG in peripheral blood mononuclear cells from patients with systemic lupus erythematosus but not from healthy controls. Ann Rheum Dis.

