# Supplementary material for: Prolactin blocks the expression of receptor activator of nuclear factor κB ligand and reduces osteoclastogenesis and bone loss in murine inflammatory arthritis
Source: Arthritis Res Ther. 2017 May 15;19:93. doi: 10.1186/s13075-017-1290-4 (PMC5433139; doi:10.1186/s13075-017-1290-4)
Supplement: Additional file 1: Table S1. — Primers used for qRT-PCR. Primers and their annealing temperatures used in quantitative RT-PCR studies. (DOCX 24 kb) [file 13075_2017_1290_MOESM1_ESM.docx]

| ***Target*** | ***Sequence (5´-3´)*** | | ***Annealing T (°C)*** |
| --- | --- | --- | --- |
| *Hprt* | Fw-TTGCTGACCTGCTGGATTAC | Rv-GTTGAGAGATCATCTCCACC | 60.2 |
| *Tnfa* | Fw-GGGCTTGTCACTCGAGTTTT | Rv-TGC CTC AGC CTC TTC TCA TT | 59.4 |
| *rIl1b* | Fw-AAAAGCGGTTTGTCTTCAAC | Rv-GGAATAGTGCAGCCATCTTT | 59.4 |
| *rIl6* | Fw-TCCAACTCATCTTGAAAGCA | Rv-TTCATATTGCCAGTTCTTCG | 59.4 |
| *Acp5* | Fw-ATTATGGGCGCTGACTTCAT | Rv-AATTTGTGCCGAGACATTGC | 59.1 |
| *rMmp9* | Fw-ACTAAGGCTCCTCTTTTGCT | Rv-ATTGGTTCGAGTAGCTGGTA | 56.2 |
| *rCtsk* | Fw-GGGCAGGATGAAAGTTGTAT | Rv-ATTATCACGGTCGCAGTTTT | 53.7 |
| *rTnfrsf11* | Fw-TCGTTAAAACCAGCATCAAA | Rv-TCCTCCAACGTTTATGGAAT | 53.7 |
| *rTnfrsf11a* | Fw-TCGTTAAAACCAGCATCAAA | Rv-TCCTCCAACGTTTATGGAAT | 56.2 |
| *rTnfrsf11b* | Fw-GTGGAGGATCAAAAATGGAG | Rv-GTTTTGGGAAAGTGGTATGC | 58.3 |
| *rPrlr Long* | Fw-ATCTTCAACATGGCCATTAC | Rv-TTCTTCCTCTCCAGTCTCAA | 56.2 |
| *rPrlr Short* | Fw-GACCTGCATCTTTCCACCAGT | Rv-AGTCAAGTTCCCCTGCAT | 60.2 |
| *rIfng* | Fw-AGCACAAAGCTGTCAATGAA | Rv-TTCTTCTTATTGGCACACTC | 58.2 |
| *rIl17a* | Fw-ATTCCATCCATGTGCCTGAT | Rv-TTCAGGTTGACCTTCACGTT | 58.3 |
| *rIl21* | Fw-ATCAACGACTTGTTGGCACA | Rv-CAAATCACAGGAAGGGCATT | 53.3 |
| *rI122* | Fw-CATCAACTCCCAATGCAAAC | Rv-TTCAAGGGTGAAGTTGAGCA | 56.3 |
| *rI123* | Fw-GCTGGATTGCAGAGCAATAA | Rv-TTGCAAACAGAACTGGCTGT | 58.4 |
| *rFoxp3* | Fw-TCCAGAGTTCTTCCACAACA | Rv-TTTCATTGAGTGTCCTCTGC | 58.4 |
| *rEbi3* | Fw-TCCTTCATTGCCACTTACAG | Rv-AAGTAGGGCACTGTGGAAAA | 58.4 |
| *rIl12a* | Fw-TCTTTGATGATGACCCTGTG | Rv-CTGAAGTGCTGCATTTATGG | 58.4 |
| *rIl10* | Fw-GTCCCACTGCCTTGCTTT | Rv-CAGTCAGCCAGACCCACAT | 53.3 |
| *Tgfb1* | Fw-CTTTAGGAAGGACCTGGGTT | Rv-CAGGAGCGCACAATCATGTT | 60.2 |
|  |  |  |  |
| *mTnfa* | Fw-CATCTTCTCAAAATTCGAGTGACAA | Rv-TGGGAGTAGACAAGGTACAACCC | 60.2 |
| *mIl1b* | Fw-GTTGATTCAAGGGGACATTA | Rv-AGCTTCAATGAAAGACCTCA | 60.2 |
| *mIl6* | Fw-GAGGATACCACTCCCAACAGACC | Rv-AAGTGCATCATCGTTGTTCATACA | 60.2 |
| *mMmp9* | Fw-CTTTGAGTCCGGCAGACAAT | Rv-TTCCAGTACCAACCGTCCTT | 59.1 |
| *mCtsk* | Fw-AACAGCAAGGTGGATGAAAT | Rv-GCCTCAAGATTATGGACAGA | 56.6 |
| *m Tnfrsf11* | Fw-ACTCCATGAAAACGCAGATT | Rv-CACAATGTGTTGCAGTTCCT | 60.4 |
| *m Tnfrsf11a* | Fw-AACGGAATCAGATGTGGTCT | Rv-TACTGCAAGCATCATTGACC | 55.1 |
| *m Tnfrsf11b* | Fw-TGAGTGTTTTGGTGGACAGT | Rv-TGCTTTCACAGAGGTCAATG | 60.4 |
| *mPrlr Long* | Fw-ACACGCGCAGATCTCCTTACCA | Rv-CCCCTTCTTGCACAGCCACTT | 56.2 |
| *mIfng* | Fw-GCGTCATTGAATCACACCTG | Rv-GACCTGTGGGTTGTTGACCT | 60.2 |
| *Rora* | Fw-ACATATCCAAATCCCACCTG | Rv-TAATCTTGATGGCACACAGC | 58.4 |
| *Rorc* | Fw-CCCTGTGTTTTTCTGAGGAT | Rv-ATGATGATGGAAAGCCAGTT | 58.4 |
| *mIl17a* | Fw-GCTATTGATTTTCAGCAAGG | Rv-AAAACAAACACGAAGCAGTT | 60.2 |
| *mIl21* | Fw-GGAGGAAAGAAACAGAAGCA | Rv-CATCTTTTGAAGGAGCCATT | 58.4 |
| *mIl22* | Fw-TGACCAAACTCAGCAATCAG | Rv-TCCACTCTCTCCAAGCTTTT | 56.3 |
| *mIl23* | Fw-AAAATAATGTGCCCCGTATC | Rv-GATCCTTTGCAAGCAGAACT | 53.3 |
| *mFoxp3* | Fw-TTCACCTATGCCACCCTTAT | Rv-TGCGAGTAAACCAATGGTAG | 58.4 |
| *mEbi3* | Fw-TCCTTCATTGCCACTTACAG | Rv-ATGATTCGCTCAGCCACAAA | 61.2 |
| *mIl12a* | Fw-TCTTTGATGATGACCCTGTG | Rv-GCTGATGGTTGTGATTCTGA | 58.4 |
| *mIl10* | Fw-AGAAATCAAGGAGCATTTGA | Rv-ATTCATGGCCTTGTAGACAC | 60.2 |

# Table S1. Primers used for RT-PCR

*Hprt,* Hypoxanthine-guanine phosphoribosyltransferase*; Tnfa,* Tumor Necrosis Factor α*; Il1b,* Interleukin 1 β*; Il6,* Interleukin 6*; Acp5,* Acid phosphatase5, tartrate resistant (TRAP)*; Mmp9,* Matrix metalloproteinase 9*; Ctsk,* Cathepsin K*; Tnfrsf11,* Receptor Activator for Nuclear Factor κ B Ligand*; Tnfrsf11a,* Receptor Activator for Nuclear Factor κ B*; Tnfrsf11b,* Osteprotegerin*; Prlr,* Prolactin Receptor*; Ifng, Interferon γ; Il17a,* Interleukin 17A*; Il21,* Interleukin 21*; Il22, ­­*Interleukin 22*; Il23,* Interleukin 23; *Rora*, Retinoid-related orphan receptor alpha; *Rorc*, Retinoid-related orphan receptor gamma; *Foxp3,* Forkhead box P3; *Ebi3,* Epstein-Barr Virus Induced 3; *Il12a*, Interleukin 12a; *Il10*, Interleukin 10; *Tgfb1*, Transforming Growth Factor Beta 1.
